# Avoiding drug residues: a multivariate approach to estimating withdrawal intervals in edible tissues of goats following extra label administration of flunixin meglumine

**DOI:** 10.3389/fvets.2026.1736282

**Published:** 2026-05-07

**Authors:** Farha Ferdous Sheela, Jim E. Riviere, Majid Jaberi-Douraki, Jacqueline M. Hughes-Oliver, Ronald E. Baynes

**Affiliations:** 1 Department of Population, Health and Pathobiology, North Carolina State University College of Veterinary Medicine, Raleigh, NC, United States; 2 DATA Consortium and FARAD Program, Kansas State University Olathe, Olathe, KS, United States; 3 Department of Mathematics, Kansas State University, Manhattan, KS, United States; 4 Department of Statistics, North Carolina State University, Raleigh, NC, United States

**Keywords:** drug residues, FDA tolerance limit method, linear regression, multivariate t distribution, non-central t distribution, censoring

## Abstract

**Introduction:**

Flunixin meglumine is a non-steroidal anti-inflammatory drug (NSAID) commonly used extra-label in goats, necessitating the determination of an extended withdrawal interval (WDI) to minimize the risk of violative residues at slaughter. Current U.S. Food and Drug Administration (FDA) guidance estimates WDIs using univariate ordinary least squares (OLS) regression applied to concentrations at or above the limit of detection (LOD), defining the WDI as the time at which the upper bound of the 95% confidence interval for the 99% quantile falls below a specified tolerance. However, residue concentrations measured across multiple tissues from the same animal may be correlated, and excluding observations below the LOD may distort estimates by removing information from the terminal depletion phase.

**Materials and methods:**

We propose a multivariate linear regression (MvLR) framework that jointly models inter-tissue dependence while accommodating left-censored observations. Regression parameters are estimated using OLS and generalized least squares (GLS) under the uncensored MvLR model, and via an expectation conditional-maximization (ECM) algorithm under a censored MvLR formulation. Withdrawal intervals are computed using the multivariate t-distribution to obtain the upper limit of the 95% confidence interval for the 99% quantile across tissues. The methods are illustrated using tissue-residue data from 20 Boer goats administered flunixin meglumine at 2.2 mg/kg, with five animals euthanized at each of four post-treatment time points (24, 48, 72, and 96 h) and are further evaluated in a simulation study.

**Results:**

The simulation results indicate that the ECM-based censored MvLR approach yields stable parameter estimation and reliable WDI inference in the presence of censoring. Applying this framework to the goat residue data suggests that a withdrawal interval of at least 10 days is recommended to ensure that residues across all tissues fall below conservative safety thresholds.

**Discussion:**

These findings suggest that a multivariate censored modeling framework can improve WDI estimation by accounting for inter-tissue correlation and incorporating observations below the LOD, addressing key limitations of current univariate FDA-style approaches.

## 1 Introduction

Flunixin meglumine (FM) is a non-steroidal anti-inflammatory drug (NSAID) approved by the U.S. Food and Drug Administration (FDA) for use in specific food-producing animals, including beef and non-lactating dairy cattle, to control pain and inflammation associated with endotoxemia, respiratory disease, and acute mastitis (1). The approved dosage consists of a single intravenous (IV) administration of 1.1 to 2.2 mg/kg body weight, or two IV doses administered 12 h apart, for a treatment period not exceeding 3 days. In swine, a 2.2 mg/kg intramuscular (IM) dose is approved to reduce pyrexia associated with respiratory illness (2), and a transdermal formulation (Benamine) is authorized for topical use in cattle to alleviate fever and pain caused by foot rot (3). However, FM is not currently approved for use in small ruminants, for example, goats. Its administration in this species is considered extra label drug use (ELDU) and is regulated under the Animal Medicinal Drug Use Clarification Act (AMDUCA) (4). Under AMDUCA, ELDU in food-producing animals is only permissible when a scientifically justified extended withdrawal time (WDT), also known as withdrawal interval (WDI), is established to ensure that drug residues in edible tissues fall below established safety thresholds.

The WDI is defined as the time period required for drug concentrations in edible tissues to decline below a designated safety threshold—referred to as the tolerance in the United States (5) and the maximum residue limit (MRL) in the European Union (6). It is statistically determined as the time at which the upper bound of the 99% tolerance interval, calculated with 95% confidence, falls below the specified limit, thereby ensuring that 99% of treated animals will have compliant residue levels with 95% confidence. As FM, although frequently used in food-producing animals, is not approved for use in goats; consequently, any detectable residue in goat tissues at slaughter constitutes a regulatory violation under the zero-tolerance policy. Given its widespread use, FM administration has been associated with violative tissue residues, posing significant public health risks when concentrations exceed established limits (7–10). According to the USDA Food Safety and Inspection Service's (FSIS) 2019 Red Book (11), FM was among the top five most frequently detected violative residues, with maximum positive cases reported in dairy cattle.

The FDA's tolerance limit method (TLM) (5, 12) serves as the standard procedure in the U.S. for estimating WDI in edible tissues and milk. This method applies a univariate linear regression model in conjunction with a non-central *t*-distribution to compute the upper bound of the 99% tolerance interval with 95% confidence. However, edible tissues in goats—specifically liver, kidney, muscle, and fat—exhibit physiological interdependence since their concentration is partially determined by delivery of the drug in the vascular system. This violates the assumption of independence inherent in univariate analyses. To address this, a multivariate linear regression (MvLR) model offers a more appropriate framework, as it simultaneously analyzes multiple tissue concentrations while explicitly modeling their covariance structure. Additionally, the FDA TLM recommends excluding measurements below the limit of detection (LOD) prior to analysis. The LOD represents the smallest analyte concentration that can be reliably distinguished from zero, introducing left censoring into the dataset. Ignoring such censoring can result in biased parameter estimates, particularly in pharmacokinetic and residue depletion studies, where actually very low drug concentrations are ignored in calculating the terminal depletion, resulting in unnecessarily prolonged WDI estimates. To overcome these limitations, this study proposes a multivariate modeling strategy that incorporates censoring via likelihood modification under the assumption of multivariate normality. The approach also accounts for intra-animal variability in tissue depletion trajectories, thereby improving the robustness and biological plausibility of WDI estimates.

We evaluated WDI under two distinct data-handling scenarios for observations falling below the LOD. We assume that the LOD is constant across all observations within a given tissue but may differ between tissues from the same animal. In the first scenario, such observations were excluded from the analysis to be consistent with FDA's guideline. Under this approach, parameter estimation was performed using both ordinary least squares (OLS; *MvLR*_*ols*_), which does not fully consider tissue-to-tissue correlation, and generalized least squares (GLS; *MvLR*_*gls*_), which fully considers tissue-to-tissue correlation, within a multivariate linear regression framework. In the second scenario, censored values were directly addressed using three estimation strategies: OLS (*MvLR*_*ols*_), where censored values were imputed at the LOD; GLS (*MvLR*_*gls*_), where censored values were also imputed at the LOD; and a censored multivariate regression (CMvLR) model implemented using an expectation conditional maximization (ECM) algorithm, where censoring is directly modeled to capture the probability of falling below the LOD, and tissue-to-tissue correlation is fully considered. Together these scenarios allow us to assess the influence of censoring methods and estimation techniques on WDI estimates.

The remainder of this paper is organized as follows. Section 2 describes the goat flunixin residue dataset, introduces the multivariate linear regression framework, outlines the expectation conditional maximization (ECM) algorithm for handling censored data, and presents the methodology for computing one-sided tolerance limits based on the multivariate *t*-distribution. Section 3 reports the results of the applied analysis, while Section 4 presents a simulation study designed to evaluate the performance of the proposed models. Section 5 discusses the key findings and their implications, and Section 6 concludes the paper with final remarks.

## 2 Materials and methods

### 2.1 Flunixin drug administration and tissue collection

**Figure 1:**
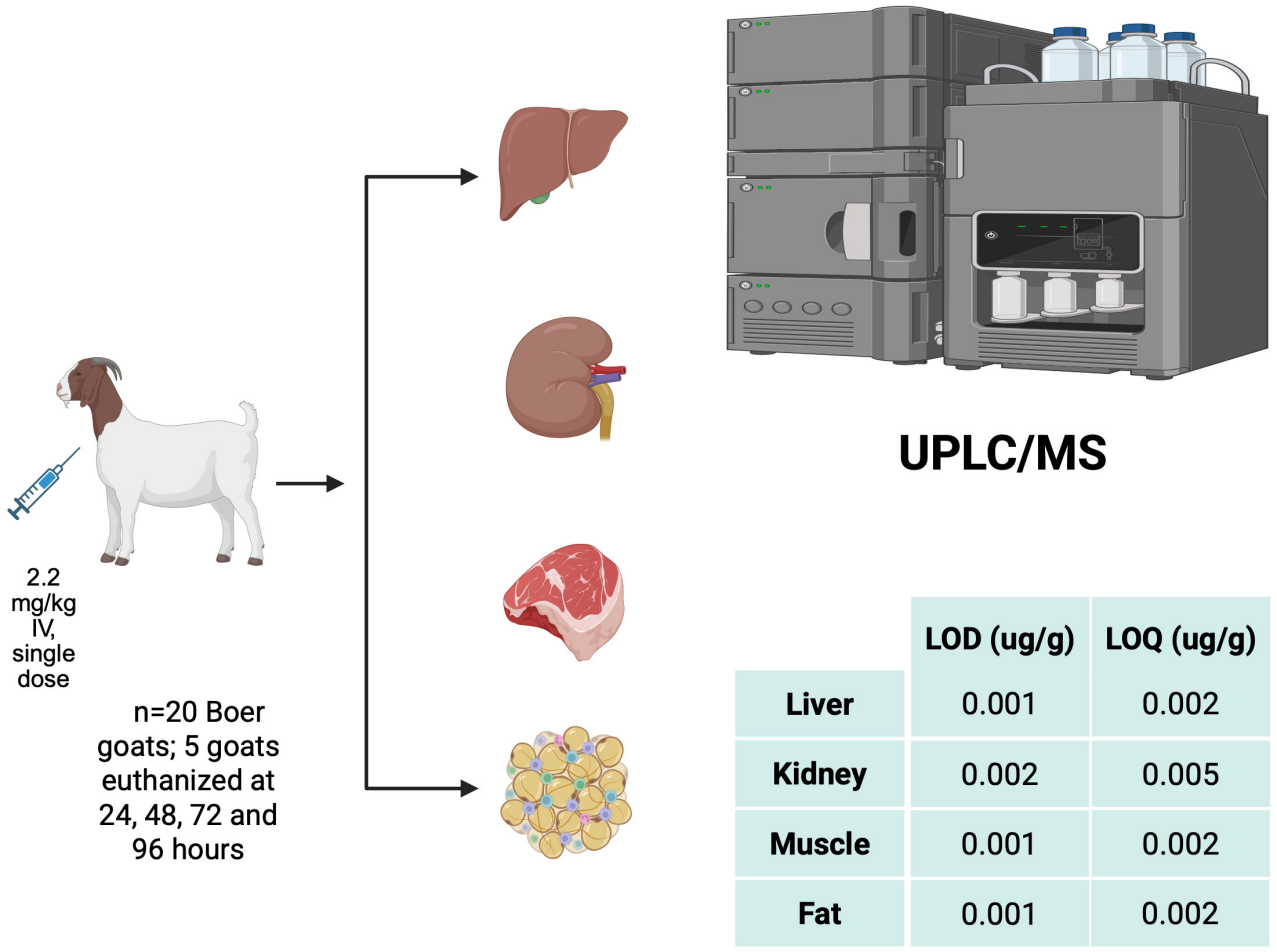
Schematic overview of the study. created in BioRender. Sheela, Farha (16).

This study involved 20 Boer goats (10 wethers and 10 does), aged 5–8 months and weighing 29.2 ± 3.1 kg, each administered a single intravenous dose of flunixin meglumine (FM) at 2.2 mg/kg (Flunixiject, 50 mg/ml, Henry Schein Animal Health, Dublin, OH), adjusted to individual body weight (Figure 1). Goats were randomly assigned to euthanasia at 24, 48, 72, or 96 h post-administration (Figure 1). Each animal was euthanized by intravenous administration of pentobarbital sodium and phenytoin sodium at a combined dose of 87 mg/kg (Euthasol^®^ Euthanasia Solution, 390 mg/ml; Virbac Animal Health, Inc., Westlake, TX, USA) following intravenous sedation with 0.5 mg/kg xylazine (Rompun^®^ Xylazine Injection, 100 mg/ml; Dechra Veterinary Products, Overland Park, KS, USA). Postmortem, samples of gluteobiceps muscle, subcutaneous fat, entire liver, and both kidneys were collected for FM concentration analysis. The frozen kidney samples were processed as pre-packaged circular punches, fully homogenized to minimize variability arising from anatomical heterogeneity, and aliquoted into 0.2 g samples for analysis. Prior to freezing, liver samples were arranged to permit collection of two approximately 5 cm-diameter punches from cross-sections of each hepatic lobe (caudate, quadrate, right, and left). These were placed in Whirl-Pak^®^ bags (Whirl-Pak, Filtration Group, Chicago, IL, USA) for ease of processing and stored at −20 °C. Tissue concentrations of FM were quantified using ultra-performance liquid chromatography coupled with tandem mass spectrometry (UPLC–MS/MS; Waters Corporation, Milford, MA, USA). Detailed procedures for animal housing, drug administration, and tissue collection are described in Giles et al. (13).

#### 2.1.1 Tissue sample preparation

All tissue samples were prepared using a standardized protocol described in Giles et al. (13) and analyzed in triplicate. Approximately 0.2 g of homogenized tissue was spiked with 10 μl of the internal standard flunixin-d3 (VETRANAL^®^, MilliporeSigma, Burlington, MA, USA), equilibrated for 15 min, and extracted with 1 ml of 85:15 acetonitrile:ultrapure water containing 0.2% formic acid. Homogenization was performed using a Bead Mill 24 Homogenizer (Thermo Fisher, Waltham, MA, USA), followed by centrifugation at 10,000 × *g* for 7 min. Supernatants (800 μl) were purified by solid-phase extraction (Oasis^®^ MCX cartridges; Waters Corporation, Milford, MA, USA), eluted with 5% ammonium hydroxide in methanol, evaporated to dryness at 55 °C, reconstituted in 300 μl of 1:1 acetonitrile:water, and filtered through 0.2 μm PVDF filters (Whatman Mini-UniPrep™, Cytiva, Marlborough, MA, USA).

#### 2.1.2 Method validation and UPLC/MS conditions

Chromatographic separation was performed using Waters Ultra-Performance Liquid Chromatograph coupled to a Waters Acquity QDa mass spectrometer (Waters Corporation, Milford, MA, USA) operating in positive electrospray ionization mode, monitoring m/z 297 (flunixin) and 300 (flunixin-d3). Liver samples were analyzed using a BEH C18 column, while kidney, muscle, and fat samples were analyzed using a BEH Phenyl column. The mobile phase was a gradient. Solvent A1 was 0.1% formic acid in water. Solvent A2 was 90:10 ultra-pure water: acetonitrile. Solvent B1 was 0.1% formic acid in acetonitrile. The flow rate was 0.40 ml/min. For liver, muscle, and fat, the gradient transitioned from 70% aqueous to 90% organic over 2.5 min; for kidney, a modified aqueous–organic system was used to account for matrix effects.

Method validation followed the FDA Bioanalytical Method Validation Guidance (32). Calibration curves (1–500 ng/g) were linear (*R*^2^ = 0.99). Recovery, accuracy, and precision were determined by analyzing five replicates at low, medium, and high concentrations within the concentration range of the curve for each tissue. Intraday precision and accuracy were obtained by analyzing three different flunixin concentrations repeated five times each on the same day (see Supplementary material, section 1). Interday precision and accuracy were obtained by analyzing seven different concentrations on five different days (see Supplementary material, section 2). The limit of detection (LOD) was defined as the lowest analyte concentration producing a signal distinguishable from background noise with acceptable analytical confidence, corresponding to a signal-to-noise ratio of approximately 3:1. The limit of quantification (LOQ) was defined as the lowest concentration that could be quantified with acceptable accuracy (within ± 20%) and precision (coefficient of variation ≤ 20%), corresponding to a signal-to-noise ratio of approximately 10:1.The LOD for liver, muscle, and fat samples was determined to be 0.001 μg/g, and the LOQ was determined to be 0.002 μg/g for these tissues. The LOD for kidney samples was determined to be 0.002 μg/g, and the LOQ was determined to be 0.005 μg/g.

The analytical methods described above follow a standardized protocol previously reported for this study and are summarized here for completeness; full methodological details are available in Giles et al. (13).

#### 2.1.3 Tissue data

Table 1 summarizes the number of complete datasets for all tissue and times and the counts of observations at or above the LOD by tissue. Muscle and fat samples were unavailable for one goat (ID 9277) euthanized at 72 h, leading to its exclusion from the analysis. This yielded a complete-case dataset of *n* = 19. Table 2 presents the observations above the LOD for each tissue across all sampling times, while Table 3 shows that when restricting to cases with LOD-compliant concentrations in all tissues, the sample size is further reduced to *n* = 8. The mean flunixin concentrations in liver, kidney, muscle, and fat tissues at each post-dose sampling time are provided in Supplementary Table S1. For each tissue and time point, concentrations were first averaged within animal and then summarized as the arithmetic mean across animals.

**Table 1:** The number of complete cases and the number of observations at or above the LOD in the respective tissues for Flunixin in goats (n = 20).

Tissues	No. of mean con. above LOD	No. of complete datasets
Liver	17	20
Kidney	16	20
Muscle	9	19
Fat	13	19

**Table 2:** Concentrations at or above the LOD at time 24, 48, 72, and 96 h across the tissues.

Time	Liver	Kidney	Muscle	Fat
24	5	5	5	5
48	5	5	2	3
72	4	3	1	3
96	3	3	1	2
Total	17	16	9	13

**Table 3:** The number of observations at each time point when we simultaneously considered observations at or above the LOD across all the tissues.

Time	Liver	Kidney	Muscle	Fat
24	5	5	5	5
48	1	1	1	1
72	1	1	1	1
96	1	1	1	1
Total	8	8	8	8

Since FM is used extra label in goats, no official tolerance levels are established for this species. The FDA has only defined tolerances for swine (0.03 μg/g liver; 0.025 μg/g muscle) and cattle (0.125 μg/g liver; 0.025 μg/g muscle). The Food Safety and Inspection Service (FSIS) defines a minimum level of applicability (MLA) as the lowest validated concentration at which a residue detection method is reliable. For goat muscle, the FSIS MLA for FM is 0.0125 μg/g, which is half the tolerance permitted in cattle muscle. Although no MLA is available for goat liver, the MLA for kidney aligns with that of muscle, at 0.0125 μg/g.

To ensure food safety, we estimate WDI corresponding to the time by which drug concentrations in all tissues fall below defined safe levels. We considered three such levels: (1) the FDA tolerance for FM in cattle muscle (0.025 μg/g), (2) the FSIS MLA for goat kidney and muscle (0.0125 μg/g), and (3) the FARAD assay LOD for all tissues (0.001 μg/g). For conservative WDI estimation, the lowest threshold in log scale across all tissues is used as the target concentration.

Model assumptions, including multivariate normality, were evaluated using graphical and formal diagnostics (Supplementary Figure S4).

### 2.2 Multivariate linear regression model

Multivariate linear regression is an extension of the standard (univariate) linear regression that accounts for correlations among multiple outcomes. It models the relationship between multiple dependent (response) variables and one or more independent (predictor) variables. Let **Y**_*i*_ be a *m*-variate random vector, ${\text{Y}}_{i}^{T}=\left[Y_{i1}\;Y_{i2}\;\ldots \;Y_{im}\right]$. We observe (**Y**_*i*_, **x**_*i*_); *i* = 1, 2, …, *n* as the data where ${\text{x}}_{i}^{T}$ is a 1 × (*p*+1) vector of covariates with ones in the first column. In our application: there are *m* = 4 response variables, namely liver, kidney, muscle, and fat; *p* = 1 to denote time- post-dosage as the only covariate; *Y*_*ij*_ denotes the log concentration measured at *jth* tissue for *ith* goat.

The multivariate linear regression can then be written as, ${\text{Y}}_{i}={\text{B}}^{T}{\text{x}}_{i}+{\text{ϵ}}_{i}$ where **B** is the (*p*+1) × *m* matrix of regression coefficients and **ϵ**_*i*_ is a *m*×1 vector of random errors with *E*(**ϵ**_*i*_) = 0 and *Cov*(**ϵ**_*i*_) = **Σ**. Assuming that the error comes from a multivariate normal distribution, ${\text{ϵ}}_{i}\overset{iid}{\sim }N_{m}\left(0,\text{Σ}\right)$, we can write ${\text{Y}}_{i}\sim N_{m}\left({\text{B}}^{T}{\text{x}}_{i},\text{Σ}\right)$. The ordinary least squares (OLS) estimators for **B** and **Σ** can be obtained by,


$$\begin{array}{l} {\hat{\text{B}}}^{\left(ols\right)}={\left({\text{X}}^{T}\text{X}\right)}^{-1}{\text{X}}^{T}\text{Y}\;and\;{\hat{\text{Σ}}}^{\left(ols\right)}=\frac{{\left(\text{Y}-\text{X}{\hat{\text{B}}}^{ols}\right)}^{T}\left(\text{Y}-\text{X}{\hat{\text{B}}}^{ols}\right)}{n-p-1}\; \end{array}$$

respectively [(31), Results 7.1, 7.2] where **Y** is an *n*×*m* matrix with row ${\text{Y}}_{i}^{T}$ to represent all tissue concentrations for the *ith* sample and column ${\text{Y}}_{j}={\left[Y_{1j}\;Y_{2j}\;\ldots \;Y_{nj}\right]}^{T}$ to represent all sample concentrations for the *jth* tissue and **X** is an *n*×(*p*+1) matrix of covariates with *ith* row ${\text{x}}_{i}^{T}$.

Aitkens generalized least squares method (GLS) (14) can also be used to estimate the regression coefficients simultaneously across the entire system of equations in place of estimating the regression coefficients independently in a univariate regression model. Because **Σ** is unknown then the idea is to get an initial estimate based on OLS $\hat{\text{Σ}}$, and estimate the Aitken's generalized least squares estimators by ${\hat{\text{B}}}^{\left(g\right)}={\left[{\text{X}}^{T}\left({\hat{\text{Σ}}}^{-1}⊗{\text{I}}_{n}\right)\text{X}\right]}^{-1}{\text{X}}^{T}\left({\hat{\text{Σ}}}^{-1}⊗{\text{I}}_{n}\right)\text{Y}$ where **I**_*n*_ is a *n*×*n* identity matrix and the final estimated covariance matrix is ${\hat{\text{Σ}}}^{\left(g\right)}=\frac{{\left(\text{Y}-\text{X}{\hat{\text{B}}}^{g}\right)}^{T}\left(\text{Y}-\text{X}{\hat{\text{B}}}^{g}\right)}{n-p-1}$ (16, vol. 10, sec. 10.2.3, page 300).

### 2.3 Censored multivariate linear regression (CMvLR)

Censored multivariate linear regression (15) is a statistical modeling approach that is used to analyze multiple correlated response variables (i.e., multivariate responses) when some of those outcomes are censored—that is, only partially observed due to detection limits, measurement thresholds, or truncation. Let the observed data for subject *i* = 1, 2, …, *n* be (**Z**_*i*_, **c**_*i*_, **X**_*i*_), where ${\text{Z}}_{i}^{T}=\left[Z_{i1},\;Z_{i2},\ldots ,\;Z_{im}\right]$ is the vector of responses for the *ith* subject (e.g., drug concentrations in different tissues), ${\text{c}}_{i}^{T}=\left[c_{i1},c_{i2},\ldots ,c_{im}\right]$ is the vector of censoring indicators, and ${\text{X}}_{i}=\left({\text{I}}_{m}⊗{\text{x}}_{i}^{T}\right)\in {\mathbb{R}}^{m\times m\left(p+1\right)}$ is the design matrix for subject *i*. In censored multivariate regression, some components of **Z**_*i*_ may not be true concentrations. In our application, values below the LOD are not considered reliable, so this creates a scenario of left-censoring. In other words, left censoring occurs when there is a true concentration value, but it must be treated as unknown because the value of *Z*_*ij*_that we observe falls below the LOD.

Let the true latent response variable *Y*_*ij*_ be defined as:


$$\begin{array}{l} Y_{ij}=\left\{ \begin{array}{c} Z_{ij},\&ifc_{ij}=0\\ -\infty \leq Y_{ij}\leq L_{j},\&if\;c_{ij}=1 \end{array}\right. \end{array}$$

where *L*_*j*_ is the limit of detection (LOD) for the *j*-th response (*j* = 1, 2, …, *m*). Then,**Y**_*i*_∣(**Z**_*i*_, **c**_*i*_)~*TN*_*m*_(**X**_*i*_**B**, **Σ**, Δ_*i*_) where *TN*_*m*_ denotes a truncated multivariate normal distribution with truncation region: $\Delta_{i}=\left\{{\text{Y}}_{i}^{T}=\left(Y_{i1},\ldots ,Y_{im}\right)|U_{ij}^{\left(1\right)}\leq Y_{ij}\leq U_{ij}^{\left(2\right)},j=1,\ldots ,m\right\}$ with $U_{ij}^{\left(1\right)}=-\infty$ and $U_{ij}^{\left(2\right)}=L_{j}$ if *c*_*ij*_ = 1 which indicates censoring, and $U_{ij}^{\left(1\right)}=-\infty$ and $U_{ij}^{\left(2\right)}=\infty$ if *c*_*ij*_ = 0 which indicates no censoring.

#### 2.3.1 Complete-data log-likelihood

The complete-data log-likelihood for parameter **θ** = {**B**, **Σ**} is:


$$\begin{array}{l} \ell_{c}\left(\text{θ}|\text{y}\right)=-\frac{nm}{2}\log\left(2\pi \right)+\frac{n}{2}\log|{\text{Σ}}^{-1}|-\frac{1}{2}\overset{n}{\underset{i=\;1}{\sum }}{\left({\text{y}}_{i}-{\text{B}}^{T}{\text{x}}_{\text{i}}\right)}^{T}\\ {\text{Σ}}^{-1}\left({\text{y}}_{i}-{\text{B}}^{T}{\text{x}}_{\text{i}}\right) \end{array}$$

Since no closed-form solution exists for the maximum likelihood estimates under censoring, we adopt the expectation conditional maximization (ECM) algorithm as described by Wang (15).

#### 2.3.2 Expectation conditional maximization (ECM) algorithm

Let ${\hat{\text{θ}}}^{\left(t\right)}=\left\{{\hat{\text{B}}}^{\left(t\right)},{\hat{\text{Σ}}}^{\left(t\right)}\right\}$ denote the current estimates of the model parameters at iteration *t*.

##### 2.3.2.1 E-step

We computed the expected value of the complete-data log-likelihood $\ell_{c}\left(\text{θ}|\text{y}\right)$ conditional on the observed data {**z**, **c**} and the current parameter estimates ${\hat{\text{θ}}}^{\left(t\;\right)}$:


$$\begin{array}{l} Q\left(\text{θ}|{\hat{\text{θ}}}^{\left(t\right)}\right)=E\left[\ell_{c}\left(\text{θ}|\text{y}\right)|\text{z},\text{c},{\hat{\text{θ}}}^{\left(t\right)}\right] \end{array}$$

##### 2.3.2.2 M-step

Update the parameter estimates by maximizing the conditional expectation from the E-step:


$$\begin{array}{l} {\hat{\text{θ}}}^{\left(t+1\right)}=arg\underset{\text{θ}}{\max}Q\left(\text{θ}|{\hat{\text{θ}}}^{\left(t\right)}\right) \end{array}$$

This iterative procedure continues until the convergence criteria are met. The convergence was declared when the relative change in the observed-data log-likelihood fell below a pre-specified tolerance of ε = 10^−5^. The maximum number of iterations was set to H = 5,000.

### 2.4 One-sided tolerance limit based on multivariate t distribution

We define the one-sided tolerance limit as: $Pr\left\{\mathrm{Pr}\left({\text{Y}}_{i}\leq {\hat{\text{Y}}}_{i}+{\hat{\text{Σ}}}^{1/2}\text{k}\right)\geq P\right\}=\gamma$, where: ${\hat{\text{Y}}}_{i}={\hat{\text{B}}}^{T}{\text{x}}_{i}\sim N\left({\text{B}}^{T}{\text{x}}_{i},{\text{Σ}}_{{\hat{\text{Y}}}_{i}}\right)$ is the predicted response at the new time point *t*_*i*_ where ${\text{x}}_{i}^{T}=\left[1\;t_{i}\right]$; γ, *P*∈(0, 1) are the confidence and quantile levels, respectively; and the residual sum of squares matrix $\text{F}=\underset{i}{\sum }\left({\text{Y}}_{i}-{\hat{\text{B}}}^{T}{\text{x}}_{i}\right){\left({\text{Y}}_{i}-{\hat{\text{B}}}^{T}{\text{x}}_{i}\right)}^{T}$ follows a Wishart distribution, notationally $\text{F}\sim W_{m}\left(n-p-1,\text{Σ}\right)$, where **Σ** is an *m*×*m* covariance matrix and can be estimated by $\hat{\text{Σ}}=\frac{\text{F}}{n-p-1}$ (14, Result 7.10, page 387–395). As derived in Sheela et al. (16), an approximate tolerance factor **k** is a multivariate scaling involves the Cholesky or square root of the covariance matrix. Notationally, $\text{k}=-\sqrt{\frac{n-p-1}{\nu }}A_{i}^{\frac{1}{2}}{\text{Ψ}}^{-1}\left(1-\gamma \right)$ where $A_{i}={\text{x}}_{i}^{T}{\left({\text{X}}^{T}\text{X}\right)}^{-1}{\text{x}}_{\text{i}}$ in case of OLS and $A_{i}={\text{x}}_{i}^{T}V\left({\hat{\text{B}}}^{g}\right){\text{x}}_{\text{i}}$ in case of GLS where $V\left({\hat{\text{B}}}^{g}\right)={\left[{\text{X}}^{T}\left({\hat{\text{Σ}}}^{-1}⊗{\text{I}}_{n}\right)\text{X}\right]}^{-1}$ is the variance-covariance matrix of ${\hat{\text{B}}}^{g}$ (16, vol. 10, sec. 10.2.3, page 297–300), and Ψ(·) is the cumulative distribution function (CDF) of the multivariate *t*-distribution with mean vector, $\text{μ}=-\sqrt{\frac{\nu }{n-p-1}}A_{i}^{-1/2}{\text{K}}_{p}$ and scale matrix **I**_*m*_, where **K**_*p*_ is the *P* th quantile from the multivariate normal distribution with the scale identity matrix **I**_*m*_, with degrees of freedom ν = *n*−*p*−*m*. When *m* = 1, the proposed construction reduces exactly to Owen's univariate non-central *t*-based tolerance limit used in FDA regulatory practice. In this sense, the present approach constitutes a direct generalization of the FDA-approved univariate procedure to correlated multivariate responses.

In the multivariate setting, time-dependent uncertainty does not appear as a simple vertical widening of individual tissue-specific bands. Instead, it manifests through inflation of the joint covariance structure via the design matrix, with uncertainty increasing as predictions move away from the center of the observed time points. Specifically, the widening of the tolerance limits with time arises through the design-dependent factor $A_{i}={\text{x}}_{i}^{T}{\left({\text{X}}^{T}\text{X}\right)}^{-1}{\text{x}}_{\text{i}}$ rather than through a constant vertical offset from the regression mean, and is therefore not visually identical to the univariate FDA hyperbolic envelope. The resulting tolerance region is defined in a transformed, decorrelated scale via ${\hat{\text{Σ}}}^{1/2}$, where it corresponds to a rectangular (orthant-type) region under componentwise ordering. The use of equi-coordinate quantiles enforces simultaneous upper bounds across all response dimensions, yielding conservative joint coverage and facilitating regulatory interpretation when multiple tissues must satisfy residue thresholds concurrently. Although this construction does not yield minimum-volume tolerance regions, it provides a practical and transparent extension of existing FDA-style univariate methodology to correlated multivariate settings.

Computation of the tolerance factor is carried out using a stochastic root-finding algorithm (17), implemented via the R functions qmvt and qmvnorm. We restrict attention to equi-coordinate quantiles, such that the tolerance factor **k** is an *m*-dimensional vector with identical components. A full theoretical derivation of this approach is provided in Sheela et al. (16). Because a closed-form solution for $\hat{\text{B}}$ is not available under censored estimation, the tolerance-limit construction originally developed for OLS and GLS is extended here to the ECM framework using the corresponding fitted mean and covariance estimates.

## 3 Results

### 3.1 Removing the concentrations below the LOD

**Figure 2:**
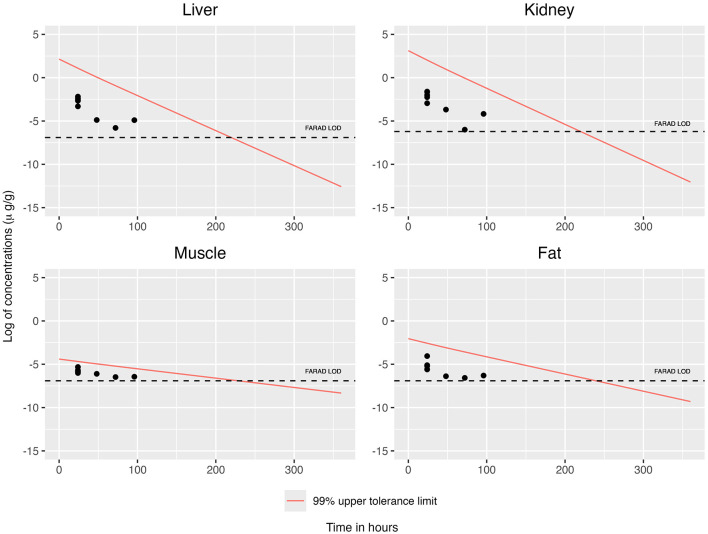
Simultaneous consideration of mean concentrations at or above the LOD across all tissues, together with the 99% upper tolerance limit at 95% confidence for each tissue using OLS.

**Figure 3:**
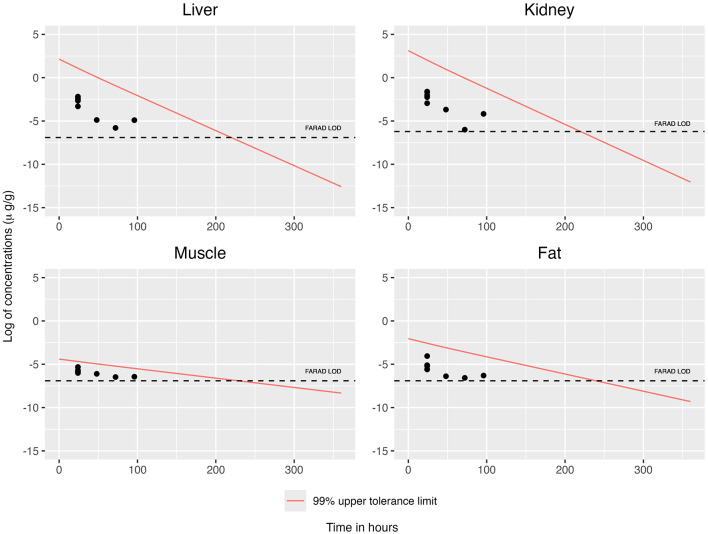
Simultaneous consideration of mean concentrations at or above the LOD across all tissues, together with the 99% upper tolerance limit at 95% confidence for each tissue using GLS.

**Figure 4:**
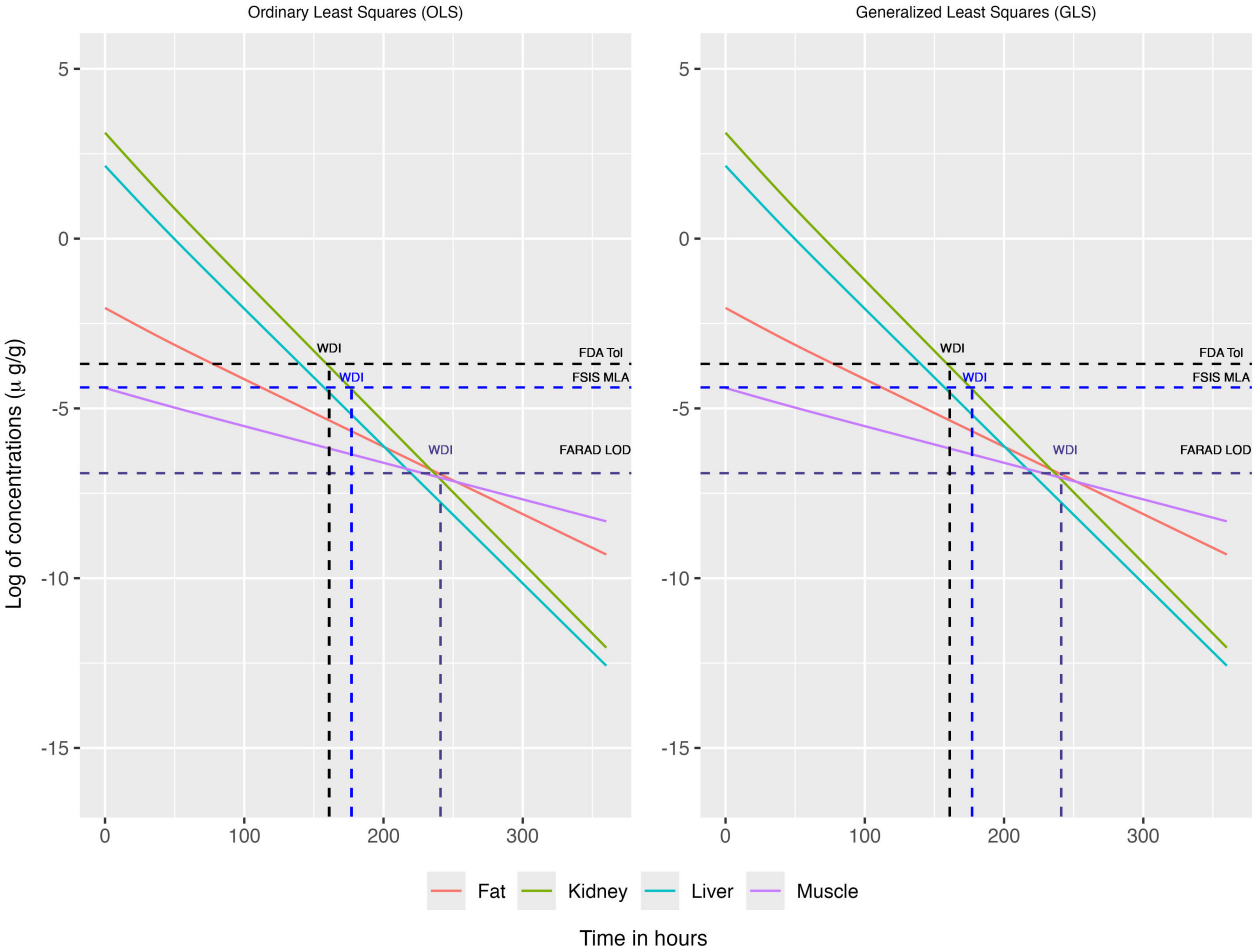
The upper 99% tolerance limit with 95% confidence at each tissue, ranging from 0 to 360 h, using OLS (Left panel) and GLS (Right panel) in the multivariate linear regression framework involving only the simultaneous consideration of mean concentrations at or above the LOD across all tissues.

When concentrations at or above the LOD were simultaneously considered across all tissues, the concentration–time profiles for each tissue are displayed in Figures 2, 3. This filtering reduces the total number of observations from n = 19 to n = 8 (see Table 3). The recommended WDI corresponds to the earliest time point at which the upper 99% tolerance limits (with 95% confidence) for all tissues simultaneously fall below their respective designated tolerances: the FDA tolerance (0.025 μg/g for muscle), FARAD LOD (0.001 μg/g), and FSIS MLA (0.0125 μg/g). Figure 4 illustrates the upper 99% tolerance limits (with 95% confidence) for all tissues across time points from 0 to 360 h.

In accordance with FDA guidance, we round up any fractional day to the next full day. Using OLS the recommended WDIs are 7, 8, and 11 days, respectively, under the FDA, FSIS, and FARAD thresholds (Table 4). Likewise, under GLS, the recommended WDIs are 7, 8, and 11 days, respectively, under the FDA, FSIS, and FARAD thresholds (Table 4). In comparison, the WDIs estimated using the FDA TLM are 8 days for liver, 15 days for kidney, 17 days for muscle, and 46 days for fat, based on tissue-specific LOD thresholds of 0.001 μg/g (liver), 0.002 μg/g (kidney), 0.001 μg/g (muscle), and 0.001 μg/g (fat) (13).

**Table 4:** Withdrawal interval (WDI) estimates in hours with rounded values in days shown in parentheses, obtained from censored and uncensored multivariate regression models.

Method	Excluding sub-LOD values (in hours)			Substituting/Imputing sub-LOD values (in hours)		
	FDA tolerance (0.025 μg/g)	FSIS MLA (0.0125 μg/g)	FARAD LOD (0.001 μg/g)	FDA tolerance (0.025 μg/g)	FSIS MLA (0.0125 μg/g)	FARAD LOD (0.001 μg/g)
OLS (MvLR)	161 (7)	177 (8)	241 (11)	139 (6)	153 (7)	264 (11)
GLS (MvLR)	161 (7)	177 (8)	241 (11)	139 (6)	153 (7)	264 (11)
Censored MvLR (ECM)	—	—	—	131 (6)	144 (6)	239 (10)

### 3.2 Handling censored data via LOD substitution and ECM

**Figure 5:**
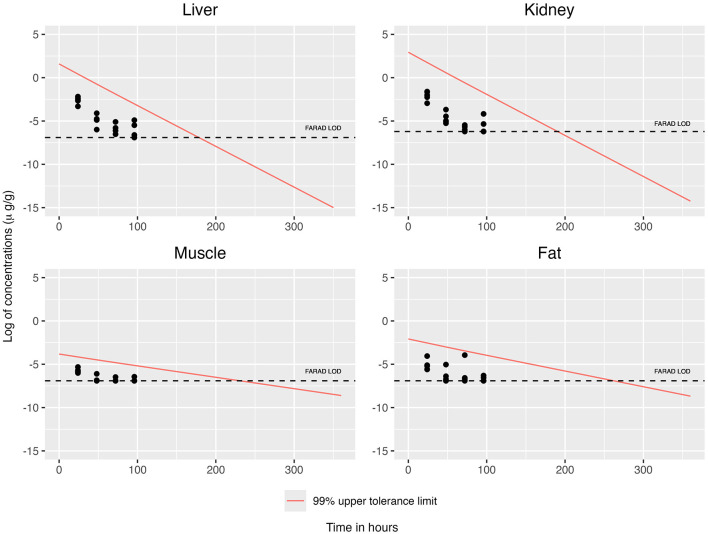
Replacing mean concentrations below the LOD by the LOD, together with the 99% upper tolerance limit at 95% confidence for each tissue using OLS.

**Figure 6:**
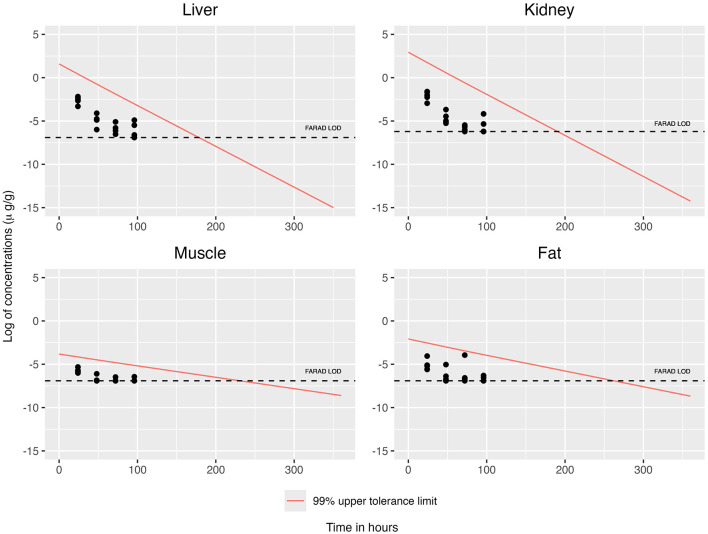
Replacing mean concentrations below the LOD by the LOD, together with the 99% upper tolerance limit at 95% confidence for each tissue using GLS.

**Figure 7:**
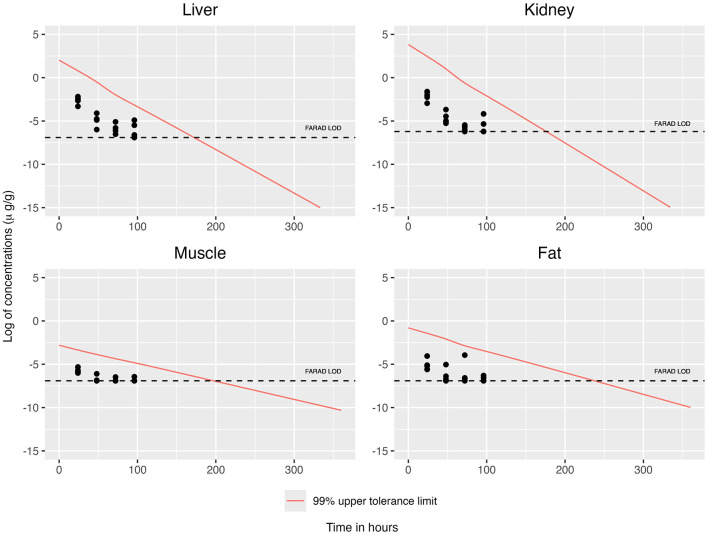
Modeling censoring using ECM, together with the 99% upper tolerance limit at 95% confidence for each tissue.

**Figure 8:**
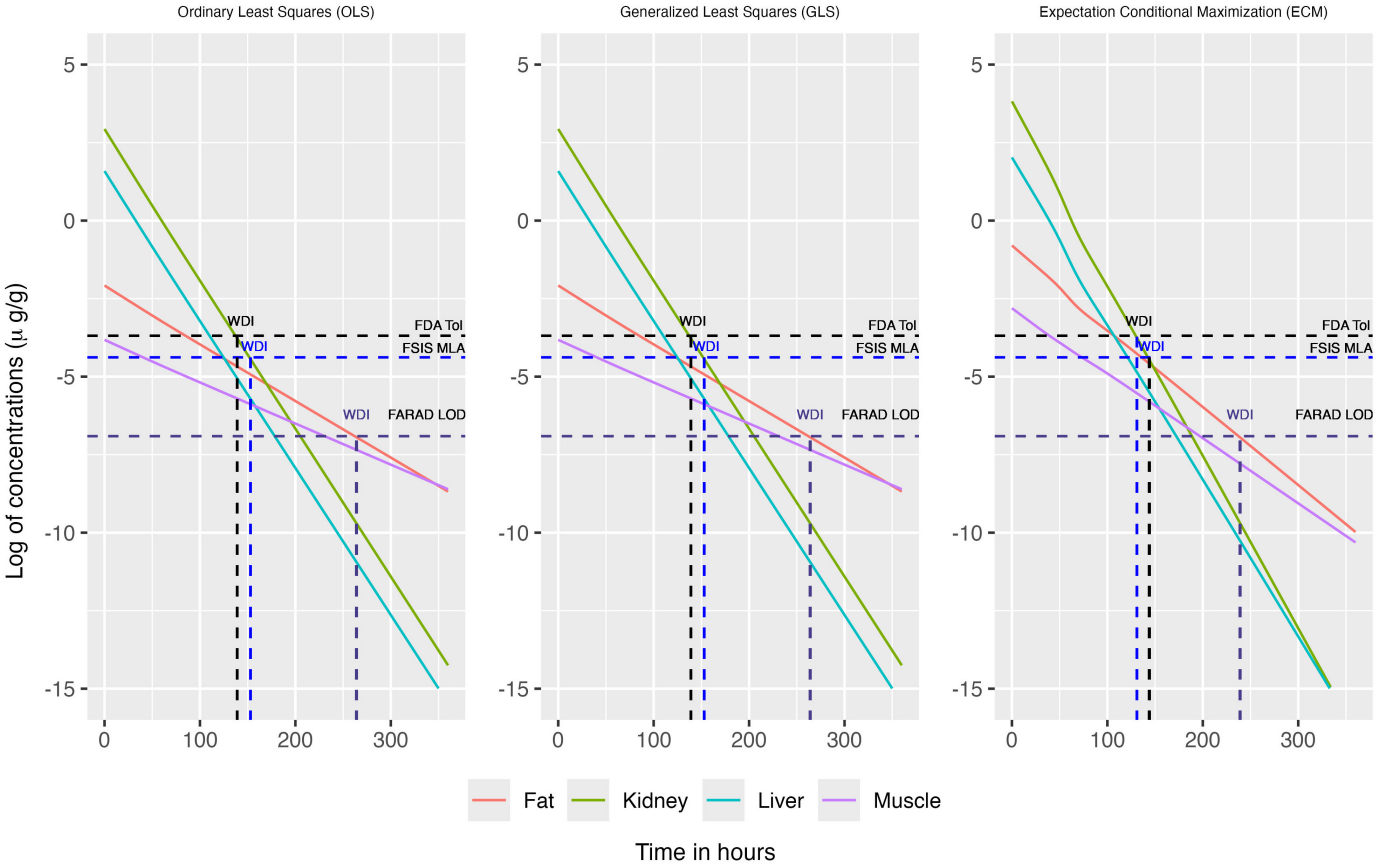
The upper 99% tolerance limit with 95% confidence at each tissue, ranging from 0 to 360 h, using OLS **(Left)**, GLS **(Middle)**, and ECM **(Right)** in the multivariate linear regression framework. In this case the mean concentrations below the LOD were replaced by the LOD of the corresponding tissue for OLS and GLS method and censoring is modeled through ECM.

For OLS and GLS estimation strategies, we substituted the corresponding tissue-specific LOD for all observations falling below the detection limits, thereby retaining the full sample size of *n* = 19 (Figures 5, 6). ECM estimation more appropriately addresses the uncertainty associated with observations falling below the LOD; by using a censoring model, it also retains the full sample size of *n* = 19 (Figure 7). Figure 8 illustrates the upper 99% tolerance limits (with 95% confidence) for all tissues across time points from 0 to 300 h.

As in the previous scenario, fractional days were rounded up to the next full day in accordance with FDA guidelines Using OLS, the recommended WDIs are 6, 7, and 11 days under the FDA, FSIS, and FARAD thresholds, respectively (Table 4). Similarly, GLS yields WDIs of 6, 7, and 11 days for the FDA, FSIS, and FARAD thresholds, respectively (Table 4). Using ECM, the corresponding WDIs are 6, 6, and 10 days under the FDA, FSIS, and FARAD thresholds, respectively (Table 4).

## 4 Simulation study

We conducted a simulation study to evaluate the performance of the expectation conditional maximization (ECM) algorithm for censored multivariate linear regression, alongside generalized least squares (GLS) and ordinary least squares (OLS), within a multivariate linear regression framework for estimating withdrawal day intervals (WDI). In both scenarios—removing or addressing concentrations below the limit of detection (LOD)—we assumed that the estimated parameters from each method from the goat data (see section 2.1) represented the true underlying parameters.

Using a multivariate normal distribution with true mean vector **XB** and variance-covariance matrix **Σ**, we generated 200 Monte Carlo datasets for each of the following sample sizes: *n* = 20, *n* = 100, *n* = 300, *n* = 500, and *n* = 1, 000. Each dataset was generated using a fixed design matrix, with the covariate matrix **X** incorporating time (0 to 800 h spaced by 24 h interval) as covariate.

For each dataset, we applied OLS, GLS, and ECM to fit the model and then computed the upper 99% one-sided tolerance limit with 95% confidence using the multivariate *t*-distribution-based tolerance limit approach. A WDI was determined for each method by identifying the earliest time point at which the tolerance limits across all tissues fell below the specified threshold. To compare the estimated WDI with the referenced true WDT, we calculated the 99% quantile of a multivariate normal distribution, treating the parameter estimates obtained from each approach (OLS, GLS, and ECM) as the true values under two distinct data-handling scenarios for observations below the limit of detection (LOD).

**Figure 9:**
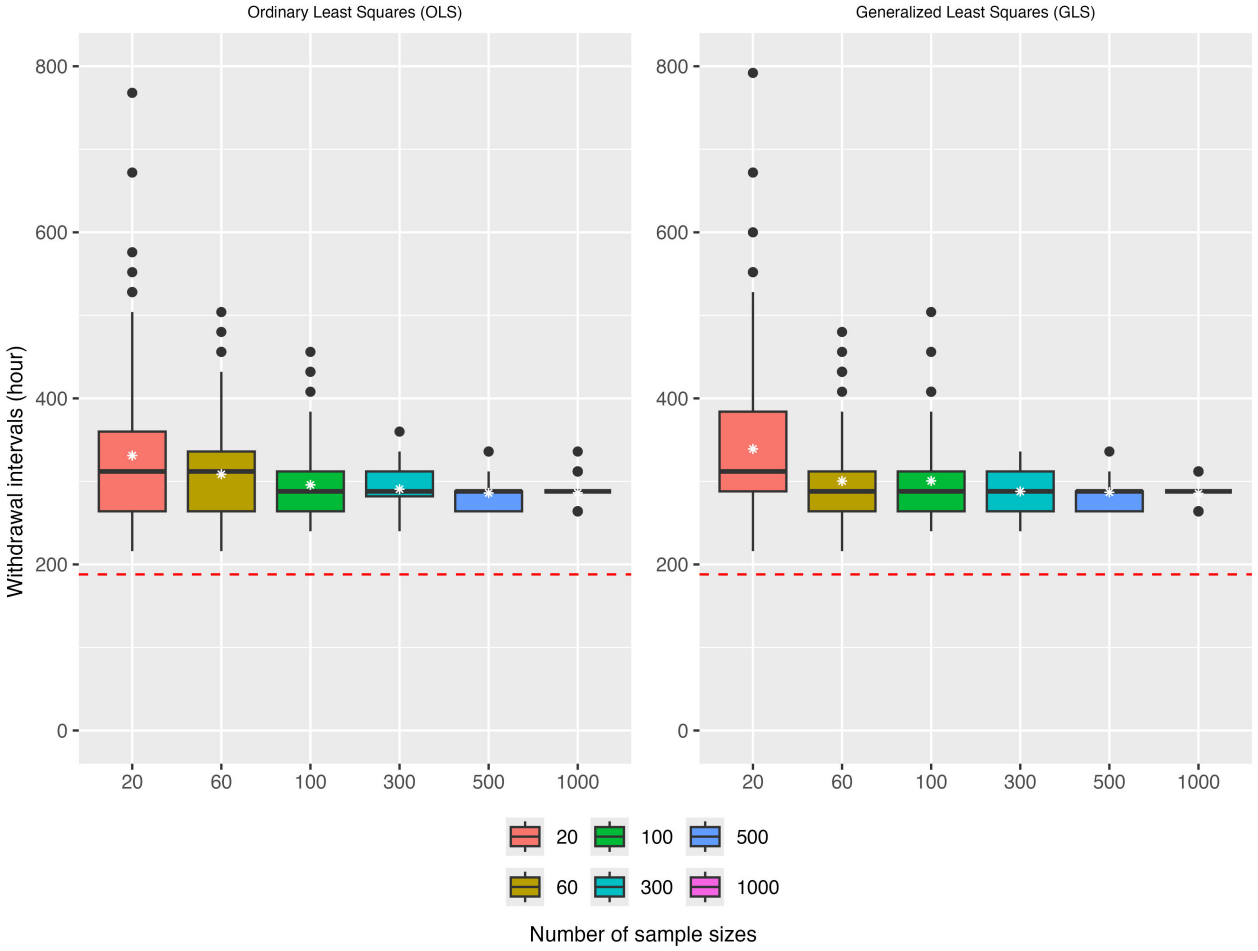
Simulation results for withdrawal intervals (WDIs) ranging from 0 to 800 h (evaluated at 24-h intervals) under the scenario where only mean concentrations at or above the LOD across all tissues were considered. The red dotted line denotes the true withdrawal time (WDT) under this simulation design.

**Figure 10:**
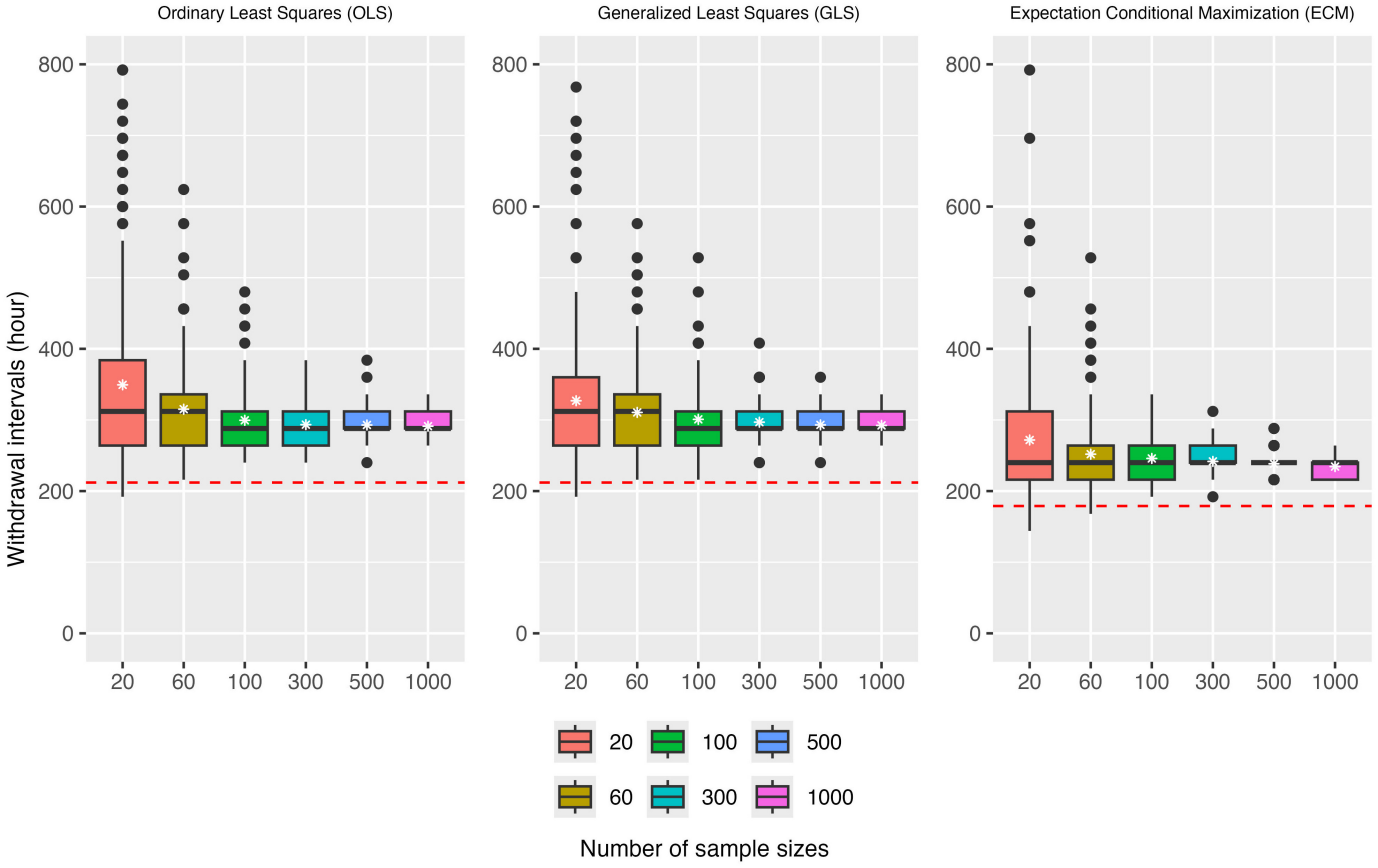
Simulation results for withdrawal intervals (WDIs) ranging from 0 to 800 h (evaluated at 24-h intervals) are shown under scenarios where sub-LOD mean concentrations were either substituted with tissue-specific LOD values for OLS and GLS or explicitly modeled as censored observations for ECM. The red dotted line denotes the true withdrawal time (WDT) under this simulation design.

For simplicity, the FARAD LOD (0.001 μg/g) was used as the sole tolerance criterion in this simulation study. The results of the simulations are presented in Figures 9, 10.

## 5 Discussion

This study presents a multivariate statistical framework for estimating withdrawal intervals (WDIs) in goat edible tissues—liver, kidney, muscle, and fat—following administration of a single extra-label intravenous dose of flunixin meglumine (2.2 mg/kg). The framework explicitly models the covariance structure among tissues and integrates left-censored residue data, arising from analytical limits of detection (LODs), directly into the likelihood function. To our knowledge, this is the first application of a censored/uncensored multivariate regression model for WDI estimation of veterinary drug residues, providing a more rigorous alternative to conventional univariate approaches.

Numerous statistical methods have been developed to handle censored responses, though most have been confined to univariate settings. Early work includes Tobin's (18) iterative maximum likelihood approach for censored data and Cohen's (19) likelihood-based estimation under normality, with Amemiya (20) establishing asymptotic properties of maximum likelihood estimators and Powell (21) proposing a censored least absolute deviations estimator. More recent contributions include Yee's (22) use of iteratively reweighted least squares (IRLS) within the vector generalized linear model (VGLM) framework, Bayesian data augmentation strategies for bivariate models by Sousa, Pereira, and Silva (23), and the expectation–conditional maximization (ECM) algorithm for censored multivariate regression developed by Wang (15). Building on these foundations, our study employs OLS and GLS within a multivariate regression setting and extends to ECM under a censored multivariate regression framework tailored to drug residue depletion. This approach simultaneously addresses left censoring and inter-tissue correlation, making it particularly advantageous in scenarios where data are sparse and censoring is substantial.

For tolerance limit construction, the FDA currently relies on Owen's (24) one-sided method under normality assumptions, which is restricted to univariate models. Extensive research has addressed univariate tolerance limits (25, 26), whereas approximate multivariate methods have been proposed by John (27), Slotani (28), Krishnamoorthy and Mathew (29), and Lee and Mathew (30). However, to remain consistent with the FDA's approved procedure, we extend Owen's univariate framework to the multivariate case by employing the multivariate *t* distribution. This allows computation of equi-coordinated one-sided upper 99% tolerance limits with 95% confidence, from which WDIs are subsequently derived under three regulatory thresholds: FDA, FSIS, and FARAD.

The empirical analysis highlights important practical implications of handling censored data in WDI estimation. Across tissues, kidney consistently exhibited the highest concentrations, peaking at 0.201 μg/g at 24 h, whereas most tissues fell below the FDA tolerance of 0.025 μg/g within 24 h. The apparent increase in mean flunixin concentrations in liver and kidney at 96 h relative to 72 h does not indicate true drug re-accumulation. Rather, it reflects cross-sectional sampling variability, as tissue concentrations at each time point were obtained from different animals rather than repeated measures. Given the small sample size per time point (*n* = 5), between-animal variability can result in non-monotonic mean patterns, particularly during the terminal depletion phase when concentrations approach the analytical limit of detection.

This effect is more pronounced in liver and kidney due to their high perfusion and central roles in drug metabolism and excretion, which are known to exhibit substantial inter-individual variability. Importantly, withdrawal interval estimation relies on regression-based modeling of depletion kinetics rather than strict monotonicity of observed means. Accordingly, these fluctuations fall within expected biological and analytical variability and do not affect the validity of the fitted depletion curves or resulting WDI estimates.

When restricting analysis to quantifiable concentrations (above the LOD), both OLS and GLS produced WDI estimates of approximately 7, 8, and 10 days under the FDA, FSIS, and FARAD thresholds, respectively (Table 4, Figure 4). Substituting sub-LOD values with LODs produced similar outcomes, yielding WDIs of roughly 6, 7, and 11 days (Table 4, Figure 8). As shown in Supplementary Figure S5, residual correlation structures were identical under ordinary least squares (OLS) and generalized least squares (GLS). Because tissue concentrations were observed at identical time points for all animals, the design matrix was common across tissues, and the data were fully balanced. Under these conditions, OLS and GLS yield equivalent residual correlation estimates. Consequently, the joint upper-tail behavior of tissue concentrations—and therefore the estimated withdrawal interval yielded the same result.

By contrast, when censoring was explicitly modeled (Table 4; Figure 8, right panel), the expectation–conditional maximization (ECM) approach produced shorter and more conservative withdrawal interval estimates of 6, 6, and 10 days under FDA, FSIS, and FARAD thresholds, respectively. The ECM-based depletion trajectories aligned more closely with the censoring-adjusted likelihood, reducing bias associated with substitution-based handling of censored observations while still accounting for tissue-to-tissue correlation. Across all modeling approaches, kidney remained the limiting tissue, whereas muscle consistently exhibited the most rapid depletion.

Together, Figures 4, 8 demonstrate that while OLS and GLS yield similar WDIs under substitution, they risk bias when censoring is extensive, whereas ECM provides more robust and conservative estimates by directly incorporating censoring into the model. Although the plotted tolerance limits on both Figures 4, 8 are approximately parallel on the log scale, this visual impression reflects presentation choices rather than time-invariant uncertainty. In particular, the log transformation compresses vertical differences at later times, and for clarity only the upper one-sided 99% tolerance limits with 95% confidence are displayed, while fitted mean trajectories for individual tissues are not overlaid to avoid visual clutter. As described in the Method section, time-dependent uncertainty is propagated through the design-matrix covariance and joint error structure, with extrapolation inflating the joint covariance ellipsoid rather than producing a simple vertical widening of individual tissue-specific bands. Consequently, widening occurs in the transformed multivariate space and need not resemble the explicit hyperbolic envelope familiar from univariate FDA tolerance-limit plots. The resulting orthant-type tolerance region enforces simultaneous upper bounds across tissues and, while not minimum-volume in a geometric sense, provides a transparent and conservative construction for joint inference when multiple responses are evaluated concurrently.

Few prior studies have investigated flunixin residue depletion in goats. Giles et al. (13) reported WDIs of 8, 15, 17, and 46 days for liver, kidney, muscle, and fat, respectively, using univariate tolerance limits and tissue-specific LODs (0.001 μg/g, 0.002 μg/g, 0.001 μg/g, and 0.001 μg/g). In contrast, our multivariate approach, which accounts for inter-tissue correlation and censoring, recommends a conservative but shorter WDI of approximately 10 days across all tissues when FARAD LOD 0.001 μg/g is used (Table 4). The discrepancy likely arises in part because previous univariate methods treated tissues independently, ignoring shared variance components that our framework explicitly captures. However, the observed difference reflects multiple contributing factors beyond the univariate vs. multivariate distinction alone.

First, the previously reported 46-day estimate corresponds to a tissue-specific WDI for fat, obtained from a univariate FDA-style tolerance-limit method applied to fat alone. In contrast, the primary WDIs reported in this manuscript are defined as the earliest time at which upper tolerance limits for all tissues simultaneously fall below the specified threshold, yielding a single cross-tissue WDI. Under our analyses, kidney—not fat—is typically the limiting tissue. We will clarify this distinction in the Discussion and present tissue-specific WDIs alongside the simultaneous cross-tissue WDI to avoid misleading direct comparisons.

Second, the univariate FDA-style analysis excludes observations below the limit of detection (LOD), which disproportionately removes late-time fat measurements and truncates the terminal depletion phase. This exclusion can flatten estimated slopes and inflate extrapolated WDIs. Our manuscript explicitly evaluates how WDI estimates change under three data-handling strategies—complete-case exclusion (*n* = 8), LOD substitution (*n*≈19), and censored multivariate regression via ECM (*n*≈19)—and we will expand the Discussion to more clearly quantify how selection induced by the simultaneous >LOD rule contributes to WDI reduction.

Third, differences in tolerance-limit construction also contribute to the observed discrepancy. The FDA univariate approach applies a non-central-t formulation independently to each tissue and expresses time-dependent uncertainty through an explicit hyperbolic scaling of the standard error. In contrast, uncertainty in the present framework arises implicitly through the design-matrix covariance associated with the fitted mean response, which increases as predictions move away from the center of the observed data. This mechanism produces widening tolerance limits without imposing a constant vertical offset from the regression line and naturally extends to the multivariate setting through the joint covariance structure. While both approaches reflect increasing uncertainty under extrapolation, the present formulation differs conceptually from the FDA hyperbolic construction and should be interpreted as an alternative statistical representation of time-dependent uncertainty rather than a direct replacement for the FDA tolerance-limit method.

Monte Carlo simulations further validated the proposed framework. Figure 9 presents result for OLS and GLS under the quantifiable-only scenario. Both methods exhibited substantial variability at small sample sizes, with extreme outliers reaching beyond 600–800 h. OLS consistently produced upwardly biased estimates of the WDI, with medians well above the true reference value (red dashed line) across all sample sizes. Although the bias attenuated as sample size increased, it persisted even at *n* = 1,000. GLS yielded slightly better estimates that were generally closer to the truth, with smaller dispersion relative to OLS at every sample size, though residual bias remained for *n*≥100. These findings indicate that while GLS improves efficiency relative to OLS, neither method fully resolves bias when only quantifiable data are available.

Figure 10 compares OLS, GLS, and ECM under the substitution/censored-data setting. Consistent with the quantifiable-only scenario, OLS produced inflated WDI estimates with high variability and numerous extreme outliers, estimates showing downward to upward trend as sample size increases. GLS again slightly improved efficiency and reduced dispersion, but estimates remained biased upward for small to moderate *n*. In contrast, ECM delivered the most accurate and stable performance: mean estimates were much closer to the true WDT across all sample sizes, and variability declined sharply as *n* increased. By *n* = 500 and 1,000, ECM estimates nearly converged to the truth with minimal bias and reduced spread. Together, these results highlight the advantage of ECM in handling censored observations, providing superior accuracy and efficiency compared with conventional OLS and GLS approaches.

To more clearly connect the simulation results with the empirical findings, we emphasize that the simulation scenarios were deliberately designed to mirror key features of the goat residue dataset, including small sample sizes, inter-tissue correlation, and varying degrees of left-censoring. This alignment enables direct interpretation of the simulation results (Figures 9, 10) in the context of the empirical patterns observed in Figures 4, 8 and Table 4. Specifically, the simulations show that when censoring is present, methods that ignore or naively substitute sub-LOD observations (e.g., OLS and GLS) tend to produce more variable and occasionally upwardly biased withdrawal interval (WDI) estimates relative to the true withdrawal time. In contrast, the ECM-based approach yields estimates that are both more stable and closer to the truth across a range of sample sizes.

This improvement arises because ECM explicitly incorporates the censoring mechanism into the likelihood, rather than replacing sub-LOD values with fixed surrogates. At each iteration, the method computes the conditional expectation of the unobserved (censored) concentrations given the observed data and current parameter estimates and then updates the model parameters using this completed-data representation. In doing so, ECM preserves both the uncertainty and the multivariate correlation structure of the censored observations, leading to less biased estimation of terminal-phase decline and, consequently, more reliable WDI determination. This behavior is consistent with the empirical analysis, where ECM produces slightly shorter yet more consistent WDIs (approximately 6–10 days) compared with OLS/GLS (approximately 6–11 days), reflecting more efficient use of terminal-phase information (Table 4; Figures 4, 8).

From a regulatory perspective, the proposed multivariate ECM framework can be viewed as a principled extension of the FDA tolerance-limit approach. It retains the central inferential goal—identifying the time at which the upper bound of the 99% tolerance interval with 95% confidence falls below a specified safety threshold—while extending the framework to jointly account for inter-tissue correlation and censoring due to detection limits. As illustrated in Figures 4, 8, this multivariate approach yields a single WDI that simultaneously satisfies residue constraints across all tissues, in contrast to the tissue-specific evaluations used in current FDA procedures. Accordingly, the method may be incorporated into regulatory workflows either as a complementary analysis to assess concordance with standard FDA-derived WDIs, or as a unified multivariate alternative in settings where dependence across tissues and censoring are substantial. This provides a statistically coherent and practically interpretable pathway for extending existing tolerance-limit methodologies without departing from their foundational principles.

## 6 Limitations and regulatory considerations

Despite these advances, several limitations should be acknowledged. First, when restricting analysis to observations in which all tissues are simultaneously above the limit of detection (LOD), the effective sample size for complete-case multivariate inference is small (*n* = 8). This limits precision in estimating a four-dimensional covariance structure and derived tolerance limits, and such results should be interpreted cautiously. These complete-case analyses are presented primarily to mirror FDA-recommended practice that excludes sub-LOD values and to provide a benchmark for comparison, rather than as the sole basis for inference. To mitigate these limitations, we evaluated approaches that retain the full dataset, including LOD substitution and censored multivariate regression via the expectation–conditional maximization (ECM) algorithm, which yielded more stable and biologically plausible withdrawal interval (WDI) estimates.

Second, requiring all tissues to be above the LOD simultaneously preferentially excludes later sampling times, truncating the terminal portion of the depletion curves and potentially steepening log-linear slopes. The censored multivariate regression framework directly addresses this selection bias by modeling the probability of falling below the LOD rather than excluding such observations, thereby retaining information from late-time measurements even when exact concentrations are unobserved. WDIs in this study were estimated at time points extending beyond the observed sampling window (approximately 2.5–3 times the last observation), an extrapolation that is inherent to regulatory WDI estimation but necessarily increases uncertainty. Accordingly, extrapolated WDIs should be interpreted conservatively and in the context of modeling assumptions and data limitations.

Additional limitations relate to biological and experimental factors. We modeled mean tissue concentrations, ignoring within-tissue replicate variability; mixed-effects or non-linear mixed-effects models may better capture this hierarchical structure. The dataset comprised healthy goats of similar age and weight, limiting evaluation of biological covariates such as sex, breed, or health status, and the current framework does not accommodate multiple concurrent drugs. Anesthetic and euthanasia agents may also influence measured tissue concentrations through transient physiological effects or peri-mortem redistribution, particularly at later time points near the analytical LOD. However, because these protocols were applied uniformly across animals, such effects are expected to be non-differential and unlikely to materially affect regression-based estimation of depletion kinetics or WDIs. Flunixin meglumine was administered as a single intravenous dose well before euthanasia, and tissue collection occurred only after completion of the euthanasia procedure. While anesthetic agents may transiently influence hemodynamics, there is no direct evidence that short-term exposure to xylazine or barbiturates leads to redistribution or re-accumulation of flunixin in edible tissues at the time scales examined in this study. In addition, the regression-based modeling framework used for WDI estimation mitigates the influence of peri-mortem physiological effects by focusing on population-level concentration–time relationships rather than individual terminal measurements.

Finally, the proposed multivariate censored regression framework is intended as a developmental methodological contribution, not as a validated regulatory replacement for the FDA tolerance-limit method. It highlights how joint modeling of correlated tissues and explicit treatment of censoring can materially influence WDI estimation in sparse residue-depletion datasets. Future progress will likely require population-based non-linear mixed-effects models that integrate experimental studies with larger observational datasets and biological covariates to expand the inference space beyond standard FDA designs.

## 7 Conclusions

This study presents a multivariate modeling framework that accounts for inter-tissue correlation and data censoring when estimating withdrawal intervals (WDIs) following extra-label intravenous administration of flunixin meglumine in Boer goats. By jointly modeling liver, kidney, muscle, and fat concentrations, the approach provides a more coherent characterization of residue depletion across edible tissues and illustrates how different strategies for handling observations below the limit of detection (LOD)—exclusion, substitution, or explicit censoring—can materially influence WDI estimates. Within the context and limitations of the available data, the analyses suggest that a WDI on the order of 10 days may be sufficient to ensure residues across tissues fall below conservative thresholds. However, these findings should be interpreted as developmental and illustrative rather than as a validated regulatory determination. More broadly, the results underscore the importance of multivariate structure and censoring awareness in residue depletion studies and motivate further work integrating these features into population-based and non-linear mixed-effects modeling frameworks.

## Data Availability

The raw data supporting the conclusions of this article will be made available by the authors, without undue reservation.
