# Supplementary material for: Avoiding drug residues: a multivariate approach to estimating withdrawal intervals in edible tissues of goats following extra label administration of flunixin meglumine
Source: Front Vet Sci. 2026 May 7;13:1736282. doi: 10.3389/fvets.2026.1736282 (PMC13189813; doi:10.3389/fvets.2026.1736282)

Supplementary material

S1 Mean flunixin concentrations in tissues

Table S1. Mean flunixin concentrations in liver, kidney, muscle, and fat tissues at each post-dose sampling time. For each tissue and time point, concentrations were first averaged within animal and then summarized as the arithmetic mean across animals.

Time (hour)	Liver (μg/g)	Kidney (μg/g)	Muscle (μg/g)	Fat (μg/g)
24	0.0774	0.1370	0.0033	0.0073
48	0.0086	0.0110	0.0010	0.0021
72	0.0032	0.0028	0.0079	0.0055
96	0.0029	0.0047	0.0008	0.0009

S2 Intraday precision and accuracy

2.1 Liver

Spiked Flunixin Concentration in Liver (μg/g)	Average Concentration in Liver (μg/g) n=5	Standard Deviation (μg/g)	Relative Standard Deviation (RSD%)	Average Accuracy (%)
0.002	0.0020	0.0002	10.2	99.5
0.005	0.0042	0.0001	3.1	84.7
0.010	0.0088	0.0003	3.3	88.5

2.2 Kidney

Spiked Flunixin Concentration in Kidney (μg/g)	Average Concentration in Kidney (μg/g) n=5	Standard Deviation (μg/g)	Relative Standard Deviation (RSD%)	Average Accuracy (%)
0.002	0.0017	0.0001	3.5	86.9
0.005	0.0046	0.0002	3.8	91.2
0.010	0.0096	0.0005	5.1	95.5

2.3 Muscle

Spiked Flunixin Concentration in Muscle (μg/g)	Average Concentration in Muscle (μg/g) n=5	Standard Deviation (μg/g)	Relative Standard Deviation (RSD%)	Average Accuracy (%)
0.002	0.0020	0.0001	6.4	100.0
0.005	0.0051	0.0002	3.2	101.4
0.010	0.0103	0.0004	4.3	103.1

2.4 Fat

Spiked Flunixin Concentration in Fat (µg/g)	Average Concentration in Fat (µg/g) n=5	Standard Deviation (µg/g)	Relative Standard Deviation (RSD%)	Average Accuracy (%)
0.002	0.0020	0.0001	5.7	98.1
0.005	0.0047	0.0003	5.8	94.1
0.010	0.0093	0.0003	2.8	93.5

S3 Inter-day precision and accuracy

3.1 Liver

Spiked Flunixin Concentration in Liver (µg/g)	Average Concentration in Liver (µg/g) n=5	Standard Deviation (µg/g)	Relative Standard Deviation (RSD%)	Average Accuracy (%)
0.001	0.001	0.0001	9.1	104.4
0.002	0.002	0.0002	8.9	101.5
0.005	0.005	0.0003	6.6	96.5
0.01	0.010	0.0004	4.1	99.4
0.05	0.048	0.0036	7.5	96.8
0.1	0.101	0.0055	5.5	101.3
0.5	0.500	0.0075	1.5	100.1

3.2 Kidney

Spiked Flunixin Concentration in Kidney (µg/g)	Average Concentration in Kidney (µg/g) n=5	Standard Deviation (µg/g)	Relative Standard Deviation (RSD%)	Average Accuracy (%)
0.001	0.002	0.0007	43.4	155.7
0.002	0.002	0.0003	14.2	109.2
0.005	0.005	0.0001	2.5	94.7
0.01	0.009	0.0006	6.5	90.4
0.05	0.049	0.0030	6.0	98.6
0.1	0.101	0.0051	5.1	100.6
0.5	0.515	0.0311	6.0	103.0

3.3 Muscle

Spiked Flunixin Concentration in Muscle (µg/g)	Average Concentration in Muscle (µg/g) n=5	Standard Deviation (µg/g)	Relative Standard Deviation (RSD%)	Average Accuracy (%)
0.001	0.001	0.0001	7.1	111.8
0.002	0.002	0.0001	4.3	98.2
0.005	0.005	0.0003	5.5	97.7
0.01	0.009	0.0004	4.4	94.6
0.05	0.049	0.0019	3.9	98.0
0.1	0.099	0.0032	3.3	99.3
0.5	0.502	0.0041	0.8	100.5

3.4 Fat

Spiked Flunixin Concentration in Fat (µg/g)	Average Concentration in Fat (µg/g) n=5	Standard Deviation (µg/g)	Relative Standard Deviation (RSD%)	Average Accuracy (%)
0.001	0.001	0.00003	2.3	109.8
0.002	0.002	0.00007	3.5	101.5
0.005	0.005	0.00025	5.3	94.2
0.01	0.010	0.00039	4.0	96.5
0.05	0.049	0.00136	2.8	97.0
0.1	0.101	0.00341	3.4	100.6
0.5	0.501	0.00354	0.7	100.3

S4 Assessment of multivariate normality assumption

Before applying the modeling approaches in several scenarios, the multivariate normality assumption was evaluated for the joint tissue residuals using both graphical and formal diagnostic methods. As shown in the multivariate Q–Q plot based on Mahalanobis distances (Figure S4), the observed distances closely followed the theoretical chi-square quantiles, except for a single observation exhibiting moderate deviation from the reference line. Consistent with this visual assessment, Mardia's tests showed no statistically significant evidence against multivariate normality (skewness $p = 0.061$; kurtosis $p = 0.615$). While the skewness component yielded a borderline p-value, the kurtosis component showed no evidence of tail heaviness. Taken together, these findings indicate that the assumption of approximate multivariate normality was reasonable for the residuals used in joint tissue modeling.

FigureS4 Multivariate normality of model residuals assessed using multivariate chi-square Q–Q plots based on Mahalanobis distances.

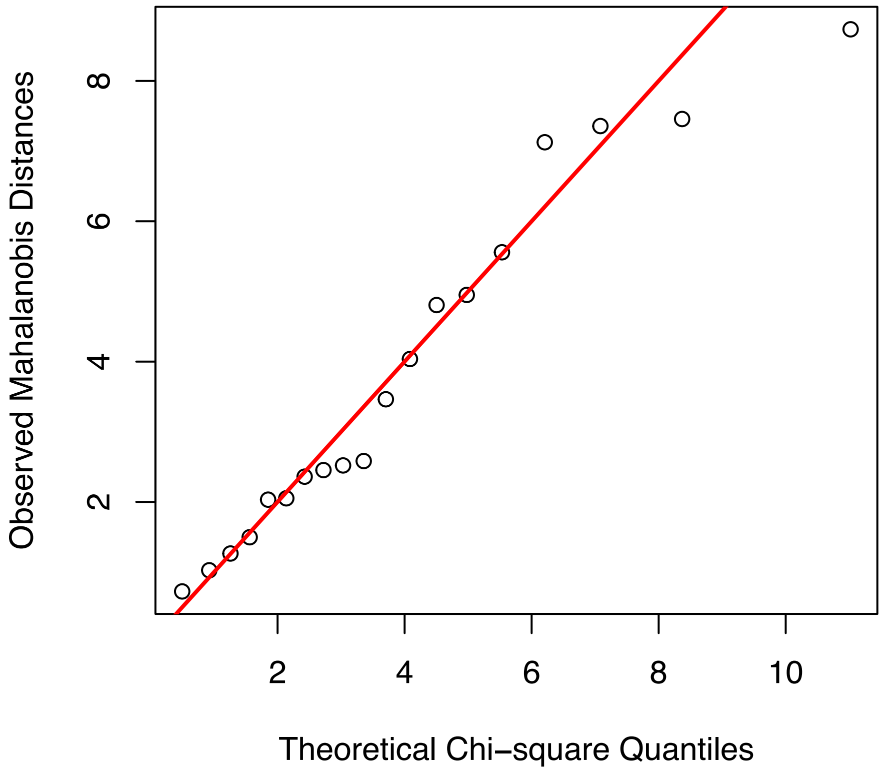

FigureS5 Heatmap of the correlations among tissues estimated using ordinary least squares (OLS) and generalized least squares (GLS).

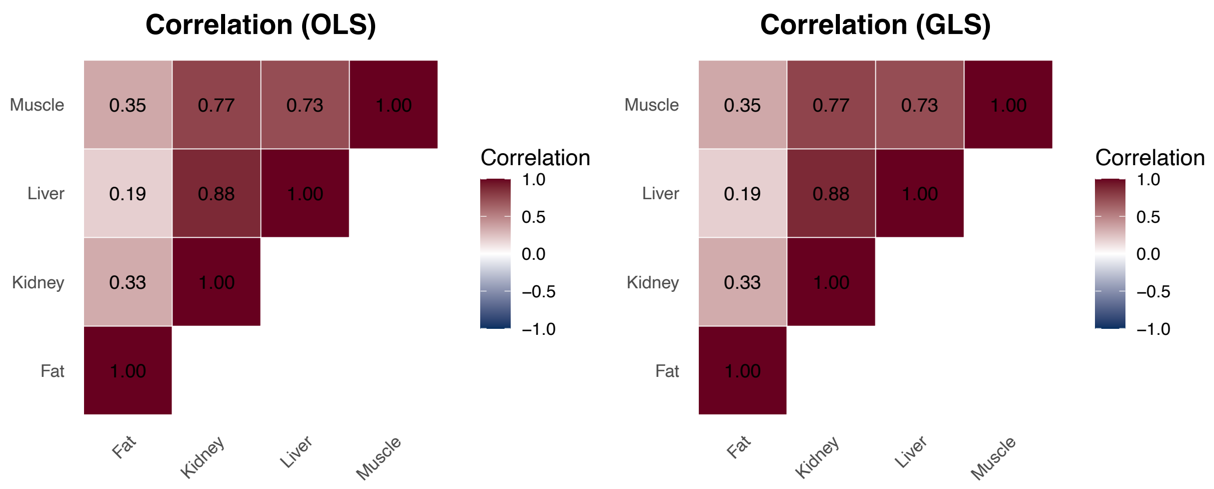

Supplement: Supplementary file 1 [file Data_Sheet_1.pdf]
